# Formal modeling and analysis of ER-*α* associated Biological Regulatory Network in breast cancer

**DOI:** 10.7717/peerj.2542

**Published:** 2016-10-20

**Authors:** Samra Khalid, Rumeza Hanif, Samar H.K. Tareen, Amnah Siddiqa, Zurah Bibi, Jamil Ahmad

**Affiliations:** 1 Atta-ur-Rahman School of Applied Biosciences (ASAB)/Healthcare Biotechnology, National University of Science and Technology, Islamabad, Pakistan; 2 Maastricht Centre for Systems Biology (MaCSBio), Maastricht University, Maastricht, Netherlands; 3 Research Center for Modeling & Simulation (RCMS), National University of Science and Technology, Islamabad, Pakistan

**Keywords:** Breast cancer, Biological Regulatory Network, Estrogen receptor-alpha, Discrete and continuous modeling, GENOTECH, René Thomas formalism, SMBioNet, Continuous Hybrid Petri Net

## Abstract

**Background:**

Breast cancer (BC) is one of the leading cause of death among females worldwide. The increasing incidence of BC is due to various genetic and environmental changes which lead to the disruption of cellular signaling network(s). It is a complex disease in which several interlinking signaling cascades play a crucial role in establishing a complex regulatory network. The logical modeling approach of René Thomas has been applied to analyze the behavior of estrogen receptor-alpha (ER-*α*) associated Biological Regulatory Network (BRN) for a small part of complex events that leads to BC metastasis.

**Methods:**

A discrete model was constructed using the kinetic logic formalism and its set of logical parameters were obtained using the model checking technique implemented in the SMBioNet software which is consistent with biological observations. The discrete model was further enriched with continuous dynamics by converting it into an equivalent Petri Net (PN) to analyze the logical parameters of the involved entities.

**Results:**

*In-silico* based discrete and continuous modeling of ER-*α* associated signaling network involved in BC provides information about behaviors and gene-gene interaction in detail. The dynamics of discrete model revealed, imperative behaviors represented as cyclic paths and trajectories leading to pathogenic states such as metastasis. Results suggest that the increased expressions of receptors ER-*α*, IGF-1R and EGFR slow down the activity of tumor suppressor genes (TSGs) such as BRCA1, p53 and Mdm2 which can lead to metastasis. Therefore, IGF-1R and EGFR are considered as important inhibitory targets to control the metastasis in BC.

**Conclusion:**

The *in-silico* approaches allow us to increase our understanding of the functional properties of living organisms. It opens new avenues of investigations of multiple inhibitory targets (ER-*α*, IGF-1R and EGFR) for wet lab experiments as well as provided valuable insights in the treatment of cancers such as BC.

## Introduction

Breast cancer (BC) is a heterogeneous disease which is one of the leading causes of cancer-related mortalities among females worldwide (DeSantis et al., 2014). Estimates indicate that out of 14.1 million new cancer cases globally (Ferlay et al., 2015), BC accounts for 25.2% of them (Hotes et al., 2004). The increasing incidence of BC is due to various genetic and environmental factors such as early menarche, late menopause, hormonal therapies, low breastfeeding, low parity and others (Madigan et al., 1995; McPherson, Steel & Dixon, 2000; Parkin & Fernandez, 2006). Increased expression of estrogen receptor-alpha (ER-*α*) is observed in 73–75% of diagnosed BC cases (Nadji et al., 2005; Rhodes et al., 2000) which leads to the disruption of various cellular processes (Seemayer et al., 2002). The mutations which increase ER-*α* expression can be caused by both genetic and environmental signals/conditions. There are two isoforms of ER, namely ER-*α* and ER-*β* (Fuqua et al., 1999; Saji et al., 2002). Approximately, there is 70% occurrence of ER-*α* positive and 30% of ER-*α* negative in the reported cases of BC (Hurvitz & Pietras, 2008; Madeira et al., 2013).

**Figure 1:**
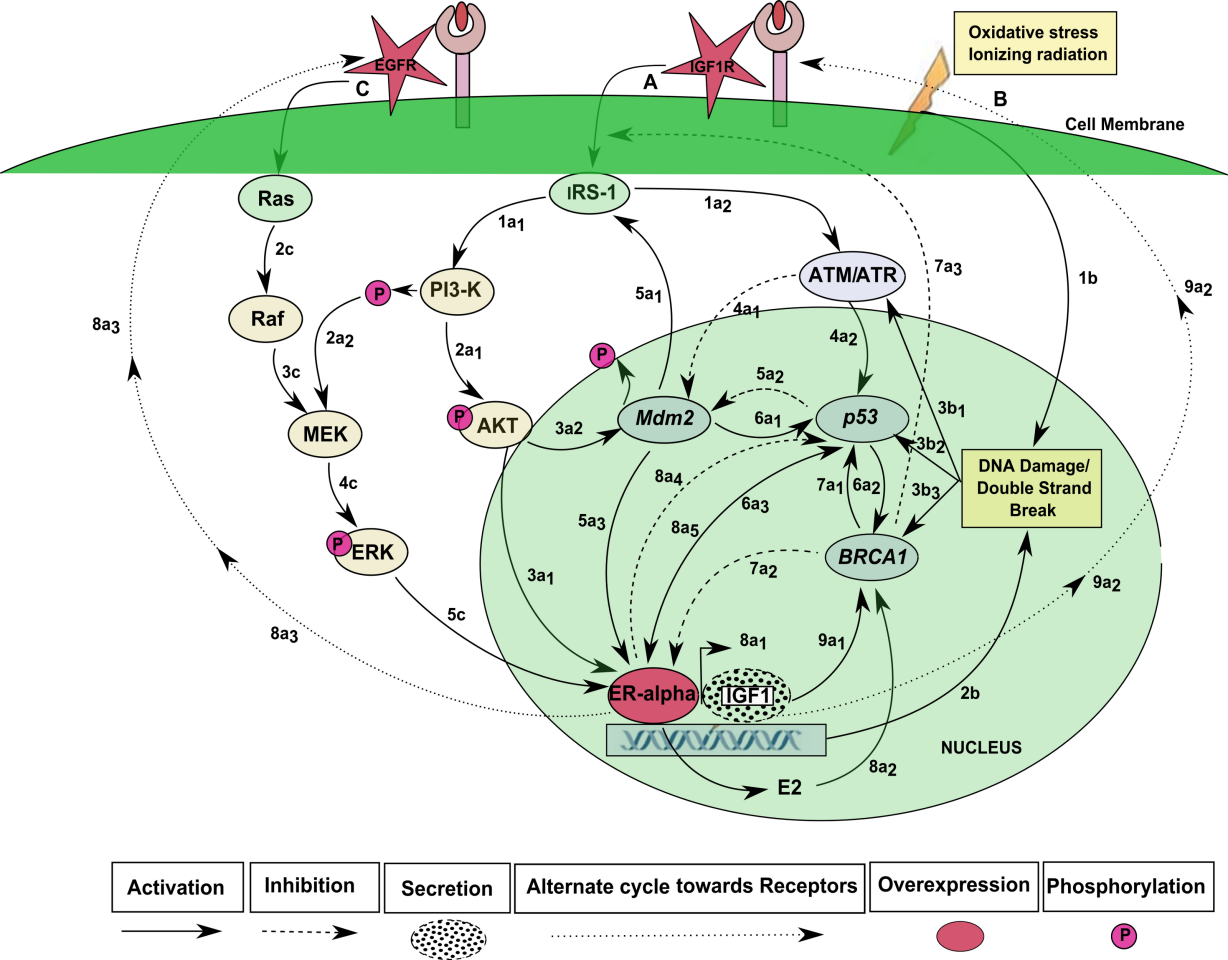
IGF-1R and EGFR signaling pathway. (A) Ligand activated Insulin growth factor receptor-1 (IGF-1R) signaling starts from the membrane to induce the insulin receptor-1 signaling. IRS-1 down-regulates the phosphoinositide-3 kinase (PI3-K) (1a_1_) which phosphorylates protein kinase B (AKT) (2a_1_). IRS-1 signaling further activates the downstream mediator Ataxia telangiectasia mutated Rad3-related (ATM/ATR) protein (1a_2_). Phosphorylated serine/threonine protein kinase (AKT) and Extracellular Signal-Regulated Kinase (ERK) signaling enhance the transactivation of estrogen receptor-alpha (ER-*α*) gene (3a1, 5c) which up-regulates the expression of insulin like growth factor-1 (IGF-1) (8a_1_). ER-*α* activates the *p*53 (8a_5_) BRCA1 gene indirectly by stimulation of estrogen (E2) in breast cells (8a_2_) and also respond to the activation of p53 gene (6a_3_). The role of ER-*α* in E2-independent manner and secreted IGF-1 mediates the over-expression of IGF-1R (9a_2_). An important role of TSG (BRCA1) also activates by the gene p53 (6a_2_). *BRCA1* suppresses the levels of ER-*α* (7a_2_) have the ability to induce apoptosis rather than cell proliferation. *BRCA1* gene can also inhibit the phosphorylation of signaling pathways of IGF-1 receptor (7a_3_). *p*53 also activates by *BRCA1* (7a_1_) which regulates the activation of *Mdm2* (6a_1_) that also suppress the over-activation of *p*53 (5a_2_). (B) There are some mutations due to radiation or oxidative stress that leads to the phosphorylation of ATM/ATR genes (1b, 3b_1_, 3b_2_, 3b_3_) and DNA damage response occurs through the increased expression of ER-*α* gene (2b) which inhibits the expression of *p*53 (8a_4_). Phosphorylated *Mdm2* expression leads to cell cycle proliferation (5a_1_) by the activation of mutated ATM/ATR signaling cascades (4a_1_). (C) An alternate pathway of ER-*α* signaling with estradiol may also utilize epidermal growth factor receptor (EGFR) for signal transduction, which may further activate the Ras, Raf protein kinases (2c, 3c). E2 causes phosphorylation of PI3-Kinase which stimulates the MEK kinase (2a_2_) and enhances the activation of extracellular-regulated kinase (ERK) (4c). In breast cancer (BC) cells the expression levels of ER-*α* is increased by phosphorylation of two receptors, IGF-1R and EGFR (8a_3_, 9a_2_).

Insulin like growth factor (IGF-1) regulates the expression of ER-*α* through the phosphoinositide-3 kinase and Serine/Threonine-Protein Kinases (PI3K-AKT) pathway which is involved in multiple mammalian cellular processes of growth and development (Ewing & Goff, 2010). Several independent studies have shown deregulation of this pathway in BC (Bailey et al., 2012; Chitnis et al., 2008; Jackson et al., 2001; Kang et al., 2012b; Kato et al., 1994; Law et al., 2008; Liu et al., 2009; Miller et al., 2005; Pollak, 2008; Riedemann & Macaulay, 2006; Sotiriou et al., 2003). The signal transduction pathway of IGF-1 regulates ER-*α* expression as shown in Fig. 1 which is constructed using literature and biological databases of interactions such as Kyoto Encyclopedia of Genes and Genomes (KEGG) (Kanehisa & Goto, 2000; Kang et al., 2012b; Levin, 2001; Pollak, 2008). The signaling cascade begins with the binding of IGF-1 to IGF-1 receptor (IGF-1R) through the phosphorylation of insulin receptor substrate-1 signaling (IRS-1) (Fagan & Yee, 2008; Law et al., 2008). It activates several downstream mediator proteins, including PI3K (Law et al., 2008; Pollak, 2008; Riedemann & Macaulay, 2006; Werner & Maor, 2006), which is involved in the activation of ER-*α* either through phosphorylation of AKT (Law et al., 2008; Pollak, 2008) or mitogen-activated kinase/extracellular signal-regulated kinase (MEK/ERK) (Watters et al., 2000).

In another pathway, MEK can also be activated by the Estrogen Growth Factor (EGF) signaling pathway, which may further activate the Ras, Raf protein kinases (Levin, 2001). IRS-1 also activates Ataxia telangiectasia mutated/Ataxia telangiectasia Rad3-related (ATM/ATR) (Law et al., 2008; Pollak, 2008; Riedemann & Macaulay, 2006) which is a serine/threonine protein kinase recruited and activated by DNA damage response (Gueven et al., 2001; Lee & Paull, 2007). ATM/ATR phosphorylates several key tumor suppressor genes (TSGs) including mouse double minute 2 homolog (Mdm2) and p53 (Werner & Maor, 2006) to regulate the transcriptional activity of *BRCA1* (Werner & Maor, 2006). Activation of *BRCA1* in oxidative stress and DNA damage response could lead to the activation of the p53 gene (Komarova et al., 2004; Schayek et al., 2009). BRCA1 and p53 genes have the ability to control cell cycle regulation (Rosen et al., 2003).

*p*53 plays an important role in the DNA damage repair detected by the enzyme ATM (Lee & Paull, 2007). In the case of phosphorylation of ATM, the expression of *p*53 is regulated by *Mdm2* (Hong et al., 2014; Powers et al., 2004). Furthermore, *p*53 is suppressed by up-regulated expression of ER-*α* which is induced by DNA damage response (Bailey et al., 2012; Liu et al., 2006; Miller et al., 2005; Sayeed et al., 2007). However, loss of function mutation of BRCA1 and p53 genes drastically increase the risk of BC and can disrupt the function of PI3K/AKT and ATM/ATR signaling (Abramovitch & Werner, 2002; Abramovitch et al., 2003; Miller et al., 2005; Vivanco & Sawyers, 2002).

Previous studies suggested ER-*α* as an important therapeutic target for the management of BC pathogenesis (Ariazi et al., 2006; García-Becerra et al., 2012; Giacinti et al., 2006; Hanstein et al., 2004; Kang et al., 2012b; Renoir, Marsaud & Lazennec, 2013; Wik et al., 2013). Although, ER-*α* is used as a drug target for the treatment of BC (Fisher et al., 1989), the underlying dynamics are far from comprehension due to the complexity of the interaction among genes/proteins involved in the signaling pathway. Preclinical studies and *in vivo* experimental strategies in cancer biology are laborious and expensive. To overcome the limitation of wet-lab experiments various Bioinformatics tools are used to study the complex regulatory networks. The computational modeling formalisms provide the dynamical insights into complex mutational diseases such as BC. In this study, we take this opportunity to study the dynamics of the IGF-1R signaling pathway by using two well-known formal computational methods, i.e., generalized logical modeling of Rene’ Thomas (Thomas, 1998; Thomas & Kaufman, 2001b; Thomas & D’Ari, 1990; Thomas & Kaufman, 2002; Thomas, Thieffry & Kaufman, 1995) and Petri Net (PN) (Brauer, Reisig & Rozenberg, 2006).

The discrete dynamics of IGF-1R/EGFR signaling was analyzed by formal modeling, which allows to study the dynamics by predicting all possible behaviors which are captured as discrete states and trajectories between them (Heinrich & Schuster, 1998). In order to construct the discrete model, we need the interaction data and threshold levels, which can be obtained through biological observations (Ahmad et al., 2006; Ahmad et al., 2012; Paracha et al., 2014). Furthermore, the continuous modelling approach applied here for the analysis of delay parameters of the IGF-1R/EGFR signalling pathway. The IGF-1R/EGFR signaling in this study implicates the down-regulation of TSGs such as BRCA1, p53 and Mdm2 in metastasis of BC. IGF-1R and EGFR should be inhibited together to control the metastatic behaviour of BC. The discrete and continuous models provide insights into possible drug targets which are captured from bifurcation states leading to both homeostatic and disease trajectories.

## Methods

**Figure 2:**
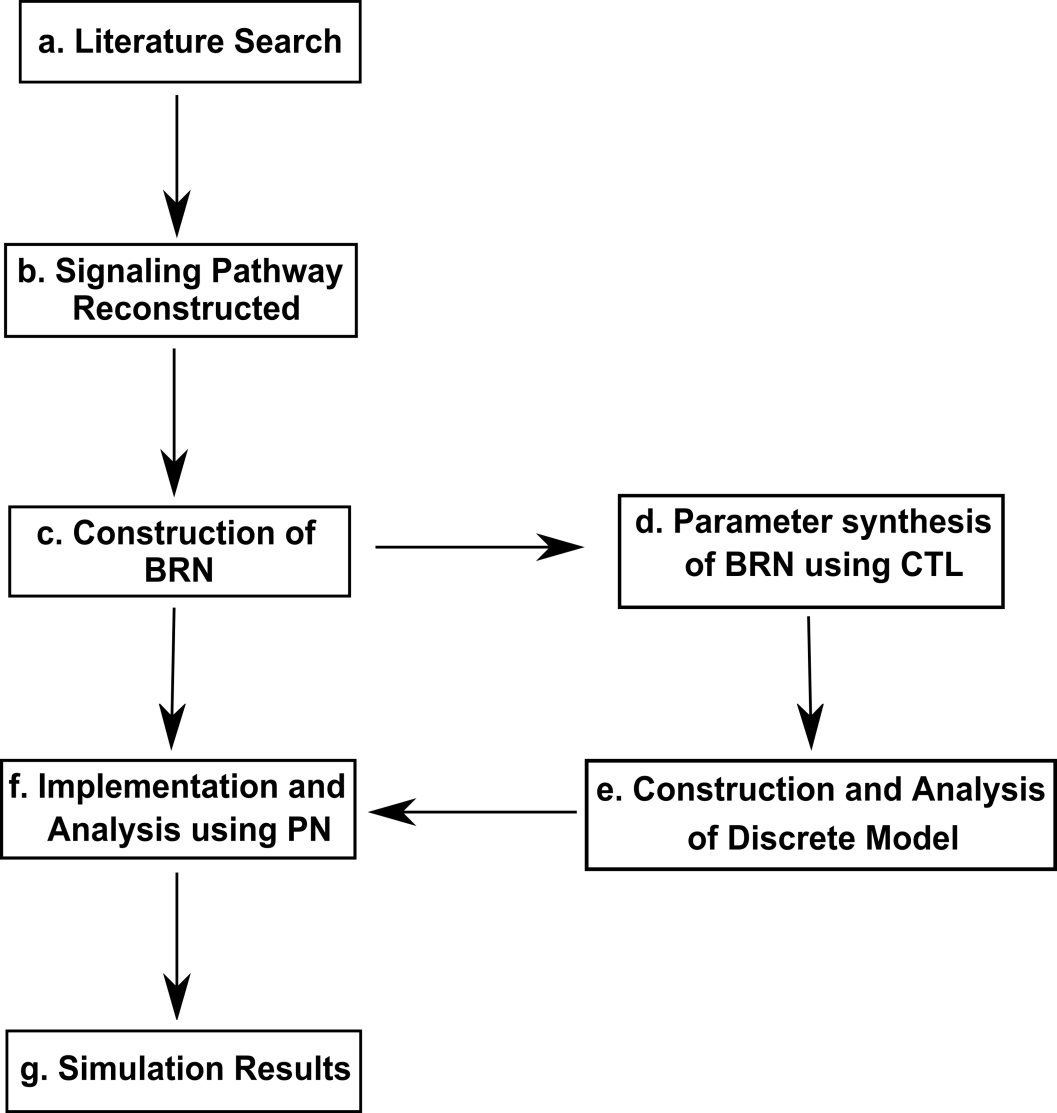
Work Flow Diagram presenting the structure and organization of the study. (A) Inference of biological observations of signaling pathways from literature survey (B) construction of interactions of proteins in the metastasis of Breast cancer (C) application of reduction approach to obtain Biological Regulatory Network (BRN) (D) parameter synthesis by using model checking method, computational tree logic (CTL) (E) analysis of the system dynamics (F) conversion of the BRN into continuous Hybrid Petri Net (HPN) (G) for simulations analysis of time-dependent dynamics.

Traditional approaches which have been used to address the complexity of biological systems include differential equations (ODEs, PDEs etc.), graph theory based formalisms (Bayesian, Logical) and fuzzy systems (De Jong, 2002). Mathematical approaches are difficult to model the complexity of non-linear dynamics of biological systems due to rare availability of system specific kinetic measures derived from expression data of biological entities. On the contrary, approaches based on Graph Theory allow to model the complexity of biological systems. The methodology for the current study is presented in Fig. 2 and explained below.

### Kinetic Logic Formalism

The kinetic logic formalism of Biological Regulatory Network (BRN) was introduced by Thomas (1973) to prove the effectiveness of discrete activity threshold levels in the analysis of the BRN as equivalent to the respective differential equations of the system (Thieffry & Thomas, 1995; Thomas, 1973; Thomas, 1981; Thomas, 2013; Thomas, Gathoye & Lambert, 1976). This method utilizes computational tree logic (CTL) formalism (Clarke, Grumberg & Peled, 1999) to detect the suitable logical parameters which can be selected through a model checker (Selection of Model of Biological Network) SMBioNet software (Bernot et al., 2004; Khalis et al., 2009; Richard, Comet & Bernot, 2006; Richard et al., 2012). These selected parameters of discrete model are abstracted from biological observations and are applied through the software, GENOTECH, to generate an asynchronous state graph (Bernot et al., 2004). A BRN consists of nodes and edges of each biological entity and transitions among them. All of the nodes are connected with edges (directed arrows) representing the activation and inhibition of node (Ahmad et al., 2012; Thomas, 1998). A dynamical network is used to determine the behavior and characterization of environmental and genetic changes in the signaling network (Thomas, 1998; Thomas & Kaufman, 2001a; Thomas, Thieffry & Kaufman, 1995).

#### Semantics of the René Thomas formalism

The semantics of the René Thomas formalism have been adapted from (Ahmad et al., 2006; Ahmad et al., 2012; Aslam et al., 2014) and are described below.

**Definition 1 (Directed Graph).**

A directed graph is represented as *G* = (*N*, *ED*), where the set of all the entities are represented by nodes, *N*, and the set of all possible transitions among entities are represented by *ED*⊆*N* × *N*. *G*^−^(*n*) and *G*^+^(*n*) represent the set of predecessors and successors nodes of a node, *n* ∈ *N*, respectively (directed from *n*1 to *n*2).

**Definition 2 (BRN).**

A BRN is a type of labeled directed graph *G* = (*N*, *ED*), representing the biological entities (genes, proteins, metabolites etc.) and the interactions amongst these entities. In a directed BRN graph each edge is pointed from tail *n*_*a*_ to head *n*_*b*_ of an edge.

1. A pair (*j*_*nanb*_, *η*_*nanb*_) is used as a label for each edge *n*_*a*_ → *n*_*b*_, where *j*_*nanb*_ is a positive integer representing a discrete threshold level and *η*_*nanb*_ represents an activation (+sign) or an inhibition (−sign).
2. The maximum number of successors of node ‘n’ is limited to *p*_*n*_ = out degree of n in which each *j*_*nanb*_ ∈ {1, 2, ……, *r*_*n*_}, where *r*_*n*_ ≤ *p*_*n*_
3. A biological entity *n* has its discrete levels in the set *Z*_*n*_ = {0, 1, …, *rn*}.

The analysis of BRN provides insight into the behavioral activity of BRN by studying the interactions between its entities to find already known or predict previously unknown behaviors.

**Types of Interactions:**

**Figure 3:**
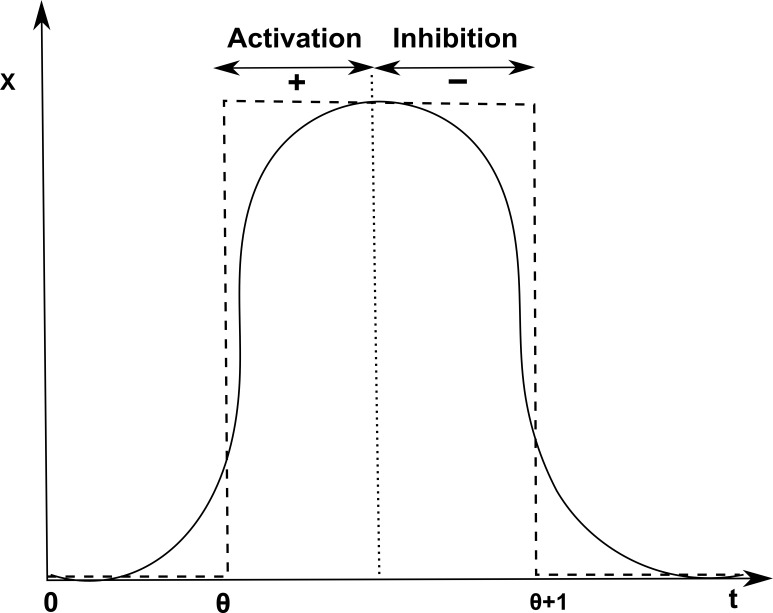
Activation and inhibition of x. Discretization of the sigmoid curve to represent activation (+) of gene x at threshold level *θ* and inhibition (−) at level *θ* + 1.

The two main types of biological regulations are in the form of activation and inhibition that represent the increase or decrease in the protein concentration respectively, shown by a sigmoid curve in Fig. 3. The activation of gene x is achieved once it reaches a level *θ* represented by positive sign “+” whereas gene x is down-regulated as it reaches threshold level *θ* + 1 represented by negative sign “−”.

**Definition 3 (Discrete States).**

A discrete state is an array of discrete levels of entities of the BRN. The state graph *G* of BRN where the discrete state is represented as a tuple *D* ∈ *S*, where;

\begin{eqnarray*}D=\prod _{na\in N}{Z}_{na} \end{eqnarray*}

and vector of discrete states defined as (*D*_*xna*_)_∀*na*∈*N*_, where *n*_*a*_ is representing the level of product *a*. A set *D* of discrete states is equal to *S* representing a directed graph in a particular configuration. The set of resources represents the presence of activators of particular entities in the absence of inhibitors.

**Definition 4 (Resources).**

Let *G* be the BRN where a set of resources *Rx*_*na*_ of a variable *n*_*a*_ ∈ *N* at a level x is considered as $R{x}_{na}=\{{n}_{b}\in {G}^{-}({n}_{a}){|}({x}_{nb}\geq {j}_{nbna}\wedge {\eta }_{nbna}=`+\text{'})\vee ({x}_{nb}\lt {j}_{nbna}\vee {\eta }_{nbna}=`-\text{'})\}$.

**Definition 5 (Logical Parameters).**

Logical parameters govern the behavior and semantics of the regulatory network. These values are represented by the equation:

\begin{eqnarray*}K(G)=\{{K}_{ni}(R{x}_{ni})\in Zn\hspace*{10.00002pt}\forall ni\in N\} \end{eqnarray*}

in which the expression level x of the entity *n* determines the set of logical parameter {*K*_*n*_(*Rx*_*n*_)}. The evolution of the level of the variable follows the following three rules:

(1) If level *x* of the entity *n* is less than *K*_*ni*_(*Rx*_*ni*_) then it increases by one discrete step, that is *x* = *x* + 1.
(2) If *x* is greater than *K*_*ni*_(*Rx*_*ni*_) then it decreases by one discrete step, that is *x* = *x* − 1.
(3) If *x* is equal to *K*_*ni*_(*Rx*_*ni*_) then it will not change, that is *x* = *x*.

It is conveniently clear from the above rules which follow the evolutionary operator ↱ (Bernot, Comet & Khalis, 2008). It tends to be evolved from one level to another for an asynchronous state graph of BRN.

**Definition 6 (Asynchronous State Graph).**

The asynchronous state graph of a BRN, where G is a directed graph which define the set of all the states and transitions of a BRN. It is represented as: *G* = (*s*, *t*), where “*s*” is a set of all states and “*t*” is *t*⊆*s* × *s* which defines the transitions among states in a directed graph. Let *O*_*xn*_ be representing the concentration level of an entity *n* in a state *Q* ∈ *s*. A state *Q* transitions to another state *Q*^∕^ iff:

1. ${Q}_{xna}\not = {Q}_{xna}^{/}$ & ${O}_{xna}^{/}={Q}_{xna}\Rsh {K}_{na}(R{x}_{na})\hspace*{1em}\exists \hspace*{1em}{n}_{a}\in N$ where ↱ represents the evolution operator (Bernot et al., 2004; Peres & Jean-Paul, 2003) and
2. ${Q}_{xnb}^{/}={Q}_{xnb}\forall {n}_{b}\in N$.

### Model checking

Model checking (Clarke & Emerson, 1982) is an exhaustive technique used to verify the existence or absence of different properties in a given system (Carrillo, Góngora & Rosenblueth, 2012). The system is represented as a state graph and different properties test for their prevalence either throughout the state graph (Carrillo, Góngora & Rosenblueth, 2012).

#### Computation of consistent network (SMBioNet)

SMBioNet (Bernot et al., 2004; Khalis et al., 2009; Richard, Comet & Bernot, 2006; Richard et al., 2012) is software used to provide the verified logical parameters of BRN by Computation Tree Logic (CTL) based model checking (Peres & Jean-Paul, 2003). CTL formulas are used to express the biological observations of the model in a model checker tool. It facilitates us by selecting only those parameter sets that are consistent with the specified CTL formulas. The selected parameters are eventually used to generate a state graph given below (see Sections ‘Isolation and selection of logical parameters’ and ‘Analysis of ER-*α* associated BRN’ for detail).

#### Syntax and semantic of CTL

The CTL algorithm is employed in the development of specification in the model that is verified by temporal logic method (Pnueli, 1977). Temporal logics encode the observations on the behavior of an entity which has interactions that tend to reach the next state in BRN. NOT (¬∕!), OR (∨∕|), AND (∧/&), implication (→), and equivalence (↔) are the logical operators employed by CTL, whereas the semantics of CTL formula are described by the temporal operators:

**Table:** 

∃ = there exist a path which starts from the current state,
∀ = All possible paths which start from the current state,
*X* = Immediate successor,
*F* = at least one state included either future or successors,
*G* = All set of states included either future or successors

For further details, a comprehensive review of syntax and semantics logical operators of CTL used in SMBioNet has been covered in detail by Khalis et al. (2009).

### Implementation of BRN in Petri Net (PN)

PN is a graph theoretical formalism which was introduced by Carl Adam for modeling of concurrent systems (Petri & Reisig, 2008). It allows intuitive representation of the system besides allowing the discrete, continuous and hybrid analysis for systemwide behaviors (Chaouiya, 2007). In this study, we have deployed PN framework to model continuous dynamics based on selected trajectories (homeostatic and pathological). It is identified by using the kinetic logic formalism based on ER-*α* associated BRN analysis. These dynamics are best specified as continuous differential equations. Our representation and analysis of the PN framework have been adapted from Chaouiya (2007); Blätke, Heiner & Marwan (2011); David & Alla (2008) are explained below.

#### Standard PN

**Figure 4:**
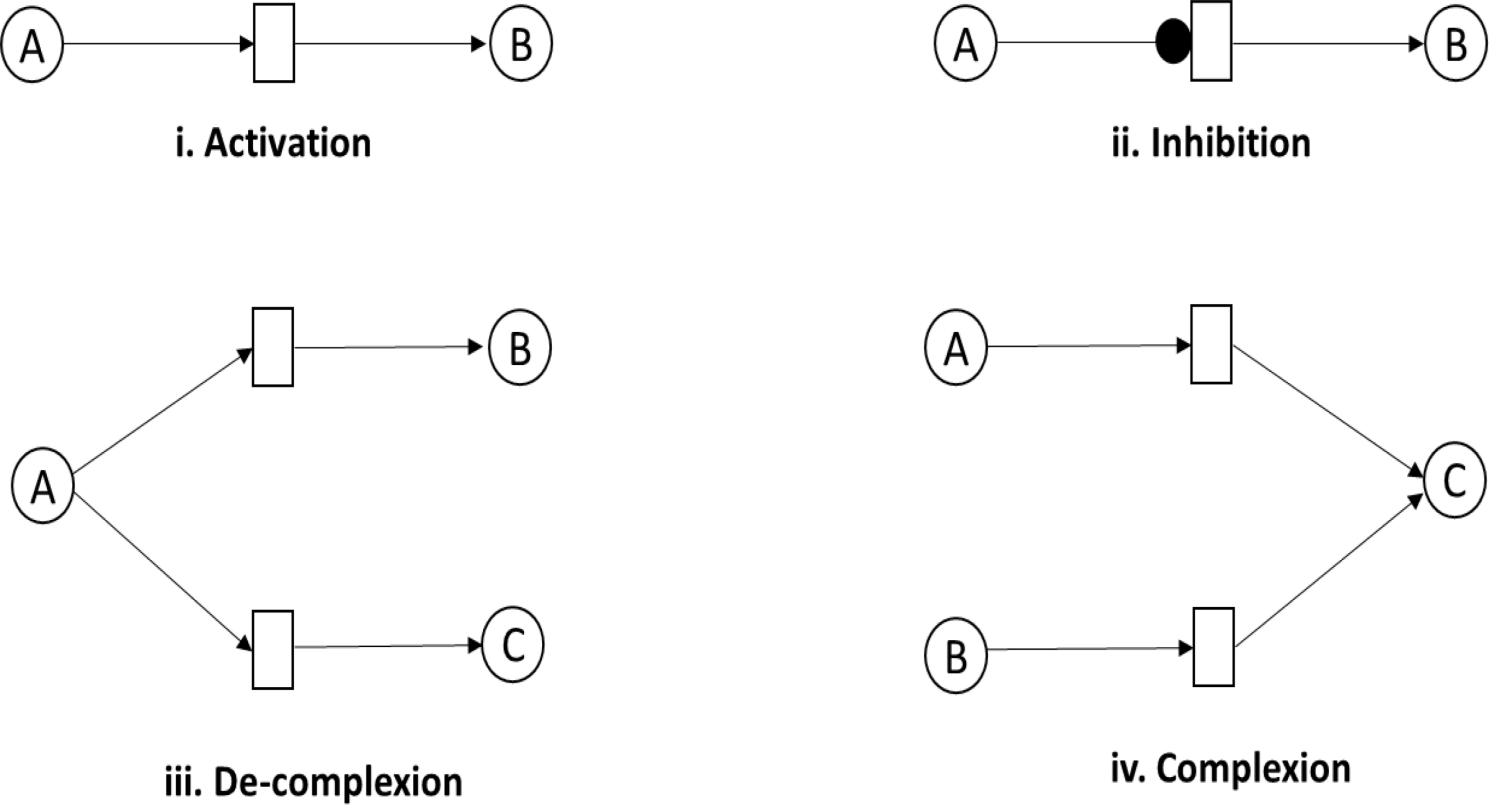
Representation of association reactions between entities. (i) Activation: entity A tends to activate another entity B (ii) Inhibition: entity A stops the activity of entity B. (iii) De-complexion process: entity A involves the activation of two entities B and C, simultaneously (iv) Complexion process: entities A and B are involved in the activation of entity C.

A PN, *N* = (*P*, *T*, *E*, *t*_0_), is a formal bipartite graph with two kinds of set of nodes represented as places *P* and transitions *T* which can be discrete in nature. The set of places *P*, drawn as circles, represents the entities such as proteins, genes and metabolites involved to design a passive part of BRN. The set of transitions *T*, represented as rectangles or squares, defines the interaction among input and out places, typically model the active part of BRN. The set of edges, *E*, defined as directed arcs are used to connect the places with transitions. These can be classified into normal, inhibitory, or test arc. An arc controls the firing in continuous process when reaction is processed from place to transition. The inhibitory arc represents the reaction where the token of input places is higher than the arc weight. A test arc is used to represent a process where the firing of transition does not change the concentration of a place such as enzymatic reactions. These biological interactions determine the dynamical behavior of entities which are involved in multiple cellular processes such as cell metabolism, differentiation, cell division and apoptosis. The marking of a place is represented by token, *t*, to describe the concentration of the entities. The firing of a transition involves the movement of tokens from pre-places to post-places. Different biological processes such as activation, inhibition, complexion, de-complexion and enzymatic reactions as represented using PN are illustrated below (Fig. 4).

#### Hybrid Petri Net (HPN)

The behavior and evolution of HPN are defined by the firing of transitions with infinite and finite number of tokens present in places. Two types of places, i.e., continuous and discrete are used to design the HPN model. In HPN (David & Alla, 2008), the infinite number of marking of continuous places is positive real numbers where the transitions fire in a continuous process while discrete places have finite numbers of tokens. HPN considers the mass action and Michaelis–Menten equations to model the firing transitions by SNOOPY (Heiner et al., 2012).

#### Petri Net model generation

In this study, we used SNOOPY (version 2.0) (Heiner et al., 2012), which is a generic and adaptive tool for modeling and simulation of graph based HPN models. We have deployed the non-parametric modeling approach which uses the token distribution within places (representing proteins) over time for monitoring the dynamics of signal flow in a signaling PN devised by Ruths et al. (2008). The concentrations of the proteins (represented as places) are modeled as tokens while their flow is represented using kinetic parameters utilizing the mass action kinetics. The value of kinetic parameter is acquired by aggregating the token count at places after each firing, which models the effect of source place on a target place. Each simulation is executed multiple times beginning with the same initial marking providing an average, signaling rate modeling the random orders of transition firings. These firing rates are able to produce the experimentally correlated expression dynamics and imitate the qualitative protein quantification techniques such as western blots, microarrays, immunohistochemistry. We used 1,000 simulation runs at 10, 50 and 100 time units for analysis. Experimental data obtained by high throughput technologies of several studies (Bailey et al., 2012; Caldon, 2014; Kang et al., 2012b; Kang et al., 2014; Liao et al., 2014; Malaguarnera & Belfiore, 2014; Moerkens et al., 2014; Cancer Genome Atlas Network, 2012; Pollak, 1998; Sotiriou et al., 2003) were used to validate the individual protein levels of the ER-*α* related BRN.

## Results and Discussion

This section explains and elaborates the results obtained from the application of the methodology and tools described in the method section.

### Construction of the ER-*α* associated BRN

**Figure 5:**
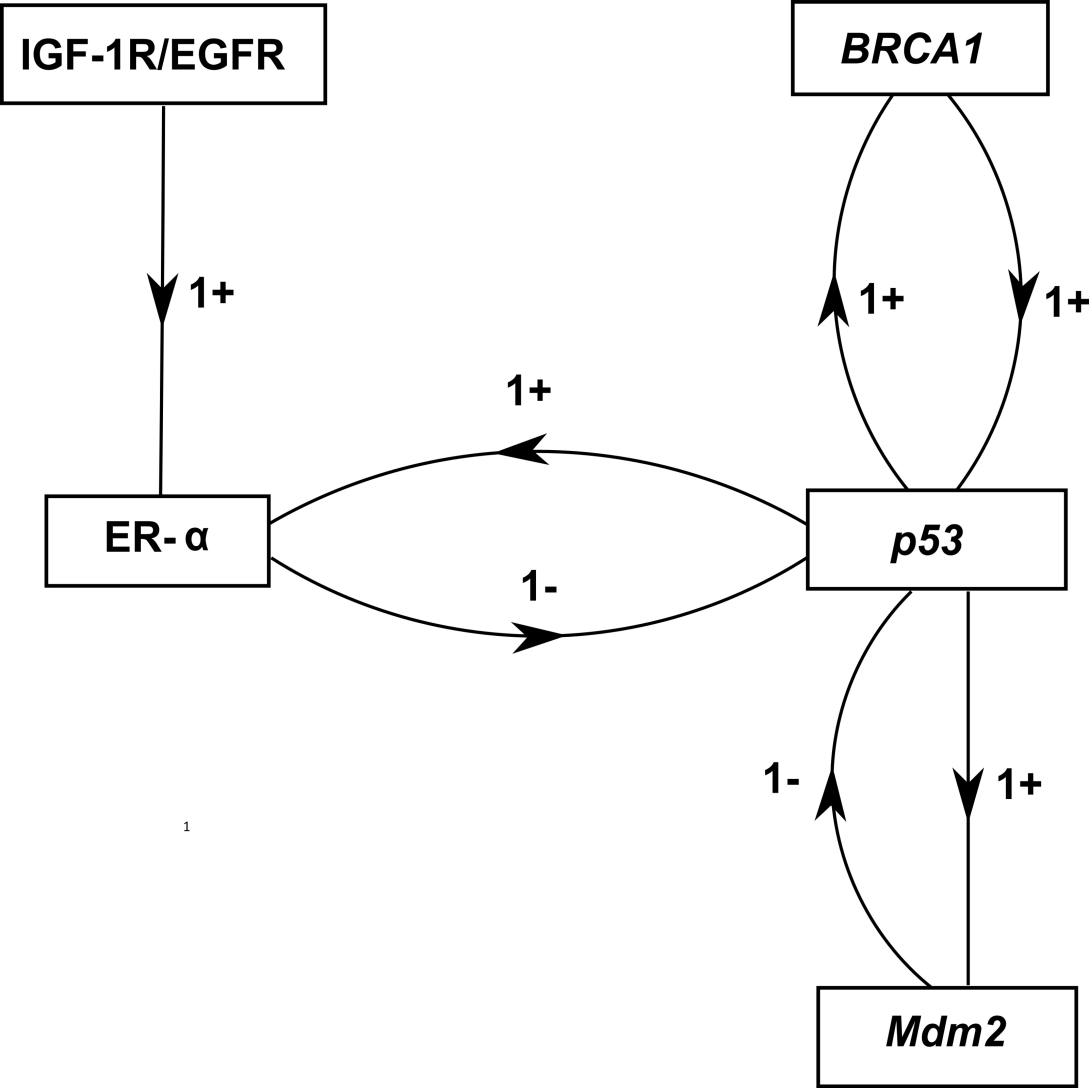
ER-*α* associated BRN. Activation is indicated by a positive (+) sign while negative (−) sign indicate inhibition. The direction of activation/inhibition is represented by arrows. The levels of entities are set according to Definition 2. The formal description of the BRN is $N=\{IGF-1R/EGFR,ER-\alpha ,BRCA1,p53,Mdm2\}$; $ED=\{(IGF-1R/EGFR\rightarrow ER-\alpha ),(ER-\alpha \rightarrow p53),(p53\rightarrow Mdm2),(p53\rightarrow BRCA1),(BRCA1\rightarrow p53),(Mdm2\rightarrow p53),(p53\rightarrow ER-\alpha )\}$.

The formal method for modeling BRN was adapted from Richard et al. (2012). The role of IGF-1R and EGFR in regulating ER-*α* was abstracted from signaling pathway shown in Fig. 1. The significance of constructing the abstracted model shown in Fig. 5 allows us to define the complex dynamical behaviors of entities which are more difficult to identify through analytical procedures, while keeping the computational complexity of the model to a minimum. We selected the key entities which interlinked at diverse points essential for behavior analysis of ER-*α* associated signaling network involved in BC. Previous studies were performed to determine the significance of TSGs in relation with over-expression of ER-*α* which is described below.

i. The interaction of ER-*α* with *p*53 mediated transcription which represents the expression levels of *p*53 (Bailey et al., 2012; Sotiriou et al., 2003).
ii. Thus, the inhibitory actions of *BRCA1* towards IGF-1R/EGFR and ER-*α* could become suppressed by the upregulated expression of ligandactivated hormonal receptor ER-*α* that is able to perform the transcriptional activation of *p*53 (Wang & Di, 2014; Yi, Kang & Bae, 2014)
iii. The TSG, p53 has positive feedback interaction with BRCA1 gene and is also involved in the activation of the Mdm2 gene (Ciliberto, Novák & Tyson, 2005; MacLachlan, Takimoto & El-Deiry, 2002; Yi, Kang & Bae, 2014).
iv. Whenever there is an increased expression of *p*53 due to some oxidative stress then it will increase the level of *BRCA1* and *Mdm2*, which will result in the respective activation or deactivation of *p*53 (MacLachlan, Takimoto & El-Deiry, 2002). Finally, the BRN was abstracted on the basis of activation of ER-*α* through loss of function mutations of TSGs such as BRCA1, p53 and Mdm2 which leads to the development of BC (Caldon, 2014).

### Isolation and selection of logical parameters

Our model of ER-*α* associated BRN has five biological entities: IGF-IR/EGFR, ER-*α*, *BRCA1*, *p*53 and *Mdm2* (Fig. 5). These biological entities have a set of discrete parameters, which represents the level of each property involved in BRN model (Table 1). Previous studies have confirmed that *BRCA1* physically interact with various transcription factors, including steroid hormone ER-*α* (Mullan, Quinn & Harkin, 2006). Active *p*53 also leads to the activation of negative regulator *Mdm2*, which acts as an inhibitor of normal function of *p*53. The discrete parameters of the constructed BRN were selected using SMBioNet by encoding the wet-lab observed behaviors in CTL. The SMBioNet analysis resulted in five sets of discrete parameters which satisfied the CTL properties, from which the fifth set was selected (given in Table 1). Its parametric values allowed closer approximation of the system, wherein gene BRCA1 must be present to stimulate p53 gene activation while ER-*α* and *Mdm2* have to be in a dormant state to allow its expression (given by parameters *K*_(*p*53),{*ER*−*α*,*BRCA*1})_ = 1, *K*_(*p*53),{*BRCA*1,*Mdm*2})_ = 1). The output file, which also shows the input model and CTL properties, is submitted along with this article as  Supplemental Information 1.

**Table 1:** List of discrete parameters of each entity of the BRN. The entities in the curly braces represent the resources available for the respective entity, whereas the number in front of the resource set represents the level which the entity will try to achieve when having that resource set.

S.No.	Biological entities	Discrete parameters
1	IGF-1R/EGFR	*K*_(*IGF*−1*R*∕*EGFR*),{})_ = 1
2	ER-*α*	*K*_(*ER*−*α*),{})_ = 0,
		*K*_(*ER*−*α*),{*p*53})_ = 1,
		*K*_(*ER*−*α*),{*IGF*−1*R*∕*EGFR*})_ = 1,
		*K*_(*ER*−*α*),{*IGF*−1*R*∕*EGFR*,*p*53})_ = 1
3	*BRCA1*	*K*_(*BRCA*1),{})_ = 0,
		*K*_(*BRCA*1),{*p*53})_ = 1
4	*p*53	*K*_(*p*53),{})_ = 0,
		*K*_(*p*53),{*ER*−*α*})_ = 0,
		*K*_(*p*53),{*Mdm*2})_ = 0,
		*K*_(*p*53),{*BRCA*1})_ = 1,
		*K*_(*p*53),{*ER*−*α*,*Mdm*2})_ = 1,
		*K*_(*p*53),{*ER*−*α*,*BRCA*1})_ = 1,
		*K*_(*p*53),{*BRCA*1,*Mdm*2})_ = 1,
		*K*_(*p*53),{*ER*−*α*,*BRCA*1,*Mdm*2})_ = 1
5	*Mdm2*	*K*_(*Mdm*2),{})_ = 0,
		*K*_(*Mdm*2),{*p*53})_ = 1

### Analysis of ER-*α* associated BRN

**Figure 6:**
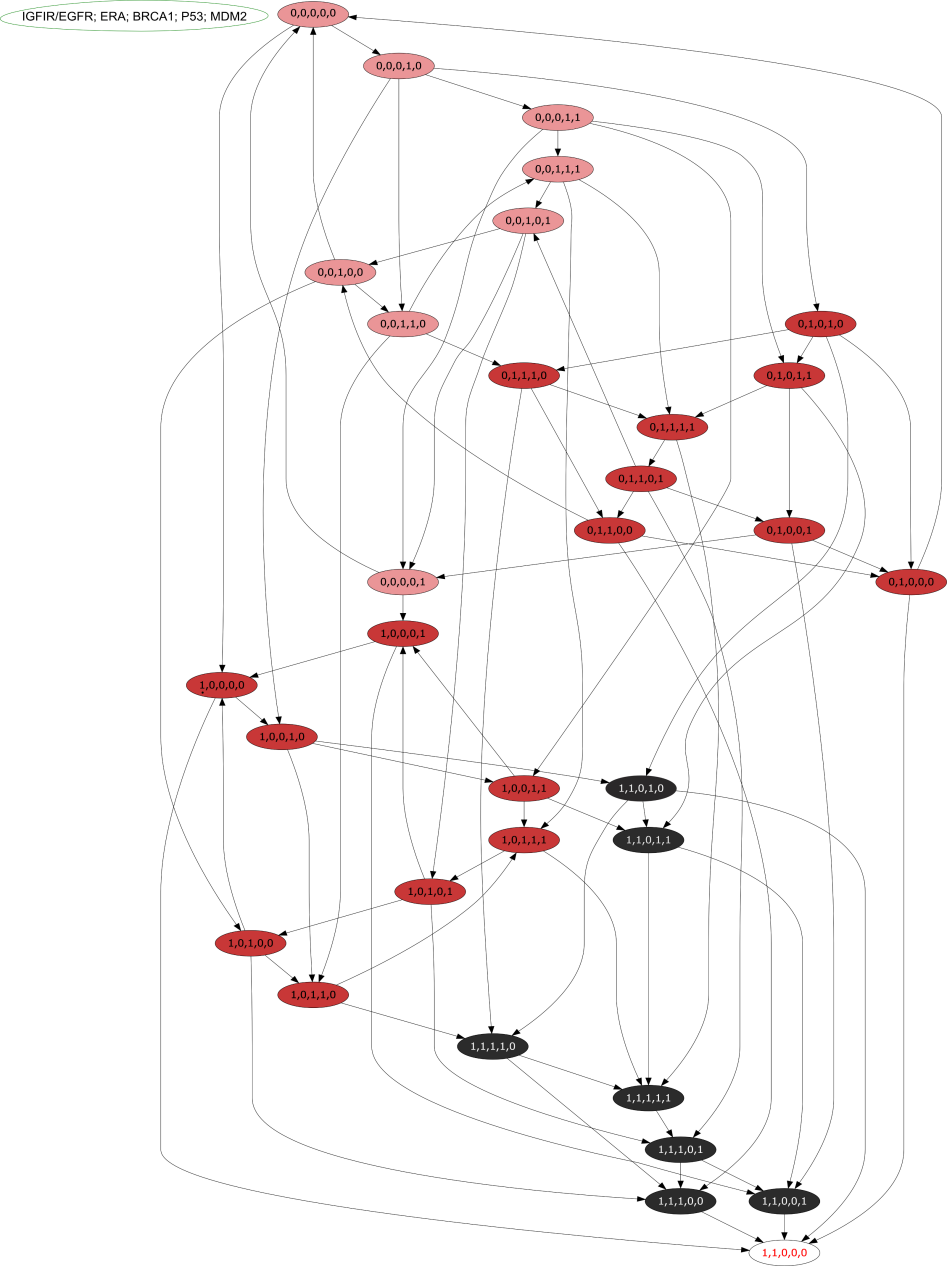
An asynchronous state graph of the ER-*α* associated BRN. The asynchronous state graph is generated by using the tool GENOTECH, utilizing the SMBioNet generated logical parameters. The initial state is taken as (0,0,0,0,0) where all entities are at their suppressed levels, whereas the deadlocked state (1,1,0,0,0) represents the metastatic state where only IGF-1R/EGFR and ER-*α* remain persistently active at cancerous levels whilst the p53, BRCA1, and Mdm2 genes are under constant suppression. The state graph is unique in the sense that it distinctly represent four zones: the pink zone (P_1_) is termed the low-risk zone since it doesn’t involve the activation of either IGF-1R/EGFR, or ER-*α*, both the proteins required for metastasis; the two red zones (P_2*a*_, P_2*b*_) are termed high risk since each zone distinctly has either IGF-1R/EGFR or ER-*α* persistently active; the black zone (P_3_) is the metastatic zone as it has both IGF-1R/EGFR and ER-*α* active, and thus leads the system towards metastasis.

The discrete parameters were then applied to the BRN using a tool GENOTECH (version 3.0) to generate the state graph shown in Fig. 6, containing the initial state (0,0,0,0,0) and the metastatic deadlocked state (1,1,0,0,0). The state graph contains 32 states, 75 unique cyclic trajectories between these states, and a distinct categorization of the 32 states into the following 4 zones (shown in Fig. 6). These zones are shown here to represent how the participating entities evolve with respect to each other’s expression level. These zones were extracted from the selected parameter set generated by the state graph. It also represents how different trajectories can arise from this BRN that could lead towards BC metastasis with up-regulated expression of IGF-1R/EGFR and ER-*α* or maintains homeostasis through the expression of *p*53, *BRCA1*, and *Mdm2*.

• **P**_1_ = (0,0,0,0,0), (0,0,0,1,0), (0,0,0,1,1), (0,0,1,1,1), (0,0,1,0,1), (0,0,1,0,0), (0,0,1,1,0), (0,0,0,0,1)
• **P**_2*a*_ = (0,1,1,1,0), (0,1,0,1,0), (0,1,0,1,1), (0,1,1,1,1), (0,1,1,0,1), (0,1,0,0,1), (0,1,1,0,0), (0,1,0,0,0)
• **P**_2*b*_ = (1,0,0,0,1), (1,0,0,0,0), (1,0,0,1,0), (1,0,0,1,1), (1,0,1,1,1), (1,0,1,0,1), (1,0,1,0,0), (1,0,1,1,0), (1,1,0,1,0), (1,1,0,1,1)
• **P**_3_ = (1,1,1,1,0), (1,1,1,1,1), (1,1,1,0,1), (1,1,1,0,0), (1,1,0,0,1), (1,1,0,0,0)

Here in Fig. 6 P_1_ (pink zone) represents a low risk zone where the levels of IGF-1R/EGFR and ER-*α* are not yet at cancerous levels. P_2*a*_ and P_2*b*_ (red zone) are high risk zones where either the level of IGF-1R/EGFR or ER-*α* is increased, but not both. The last zone P_3_ (black zone) is the metastatic zone where IGF-1R/EGFR and ER-*α* are persistently expressed. It is based on our interpretation obtained in this study through experiments not literature derived data, details of which are mentioned in the Fig. 6. The important properties based on these zones are that the 75 cycles lie within the P_1_, P_2*a*_ and P_2*b*_ zones only, with trajectories allowing passage between the zones P_1_ and P_2*a*_, but restricting P_2*b*_ to itself. The zone P_3_ on the other hand contains no cyclic trajectories. In P_3_ zone most critical state trajectories move towards a deadlock state.

The usual activation of p53 gene has been detected by the enzyme ATM (Fig. 1). It is evident from the state graph (Fig. 6) that the state (1,1,0,0,1) (in P_3_ zone) stands to be the critical most point forms where the system moves into the metastatic state (1,1,0,0,0) where all the TSGs BRCA1, p53 and Mdm2 gets suppressed. Hence, it is important to note that the system maintains a homeostatic cycle only when both IGF-1R and ER-*α* are not a co-stimulated state while other genes (BRCA1, p53 and Mdm2) remain in the oscillations. These identifications indicate that signal transduction pathway involved in the increased risk of BC progression is initiated following the activation of receptors IGF-1R and EGFR. It was concluded that IGF-1R, EGFR and ER-*α* serve as important inhibitory targets for BC treatment.

### Analysis of ER-*α* associated HPN modeling

**Figure 7:**
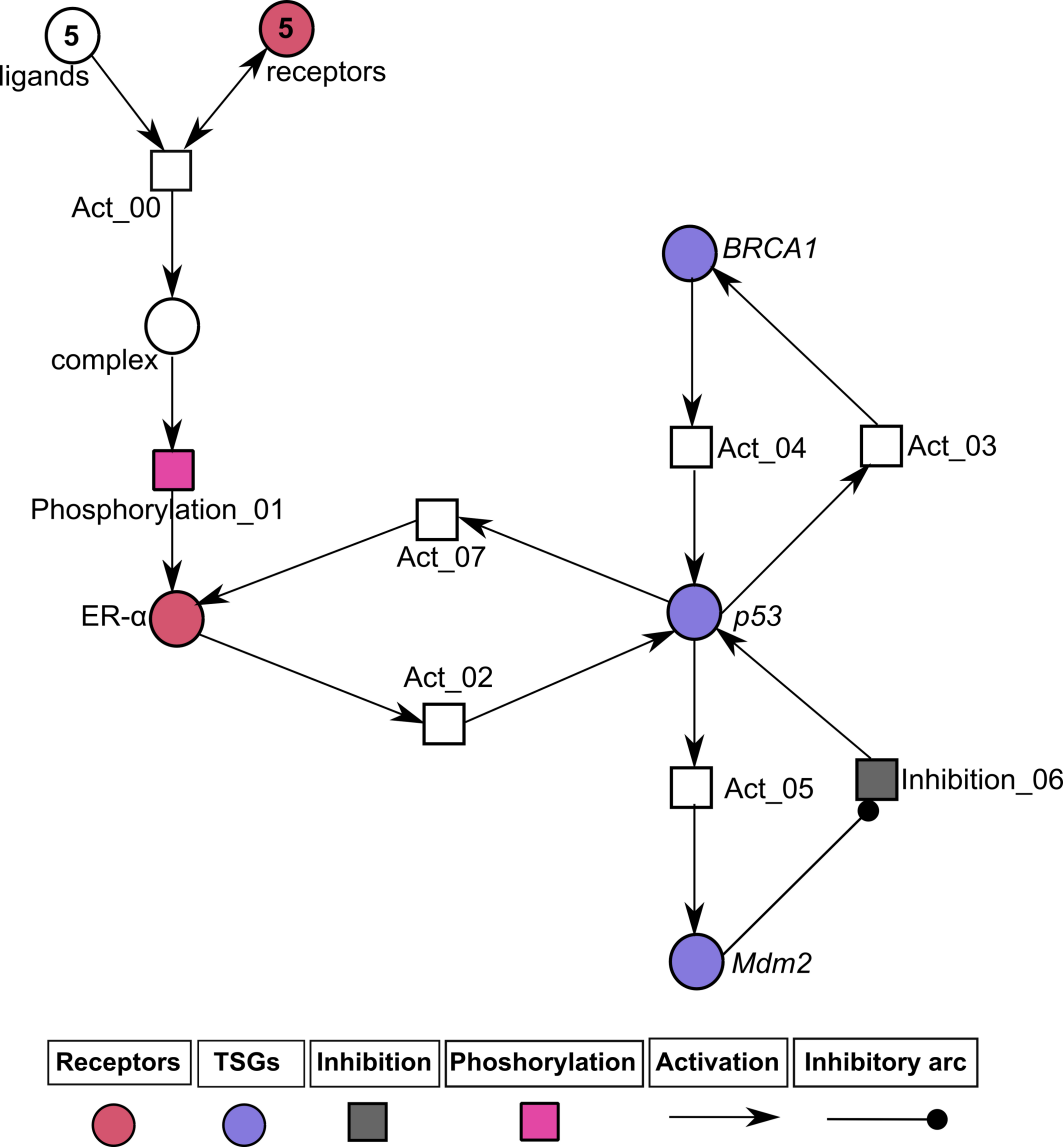
Illustration of the normal pathway of ER-*α* associated Hybrid Petri Net (HPN) model. In this PN, circles represent standard places that exhibit the behavior of ligands (IGF-1, EGF), hormonal receptors (IGF-1R, EGFR and ER-*α*) and TSGs (BRCA1, p53 and Mdm2), while the squares represent continuous transitions to demonstrate the processes of activation, inhibition and phosphorylation. Directed arrows represent activation signal coming from standard places and going towards continuous transitions. The inhibitory arc represents an inhibition signal which stops signal coming from standard places towards continuous transitions. The rate of mass action for all continuous transitions is taken as 1. The ligands (IGF-1, EGF) and the membrane receptors (IGF-1R/EGFR) are given with an arbitrary token number of 5.

**Figure 8:**
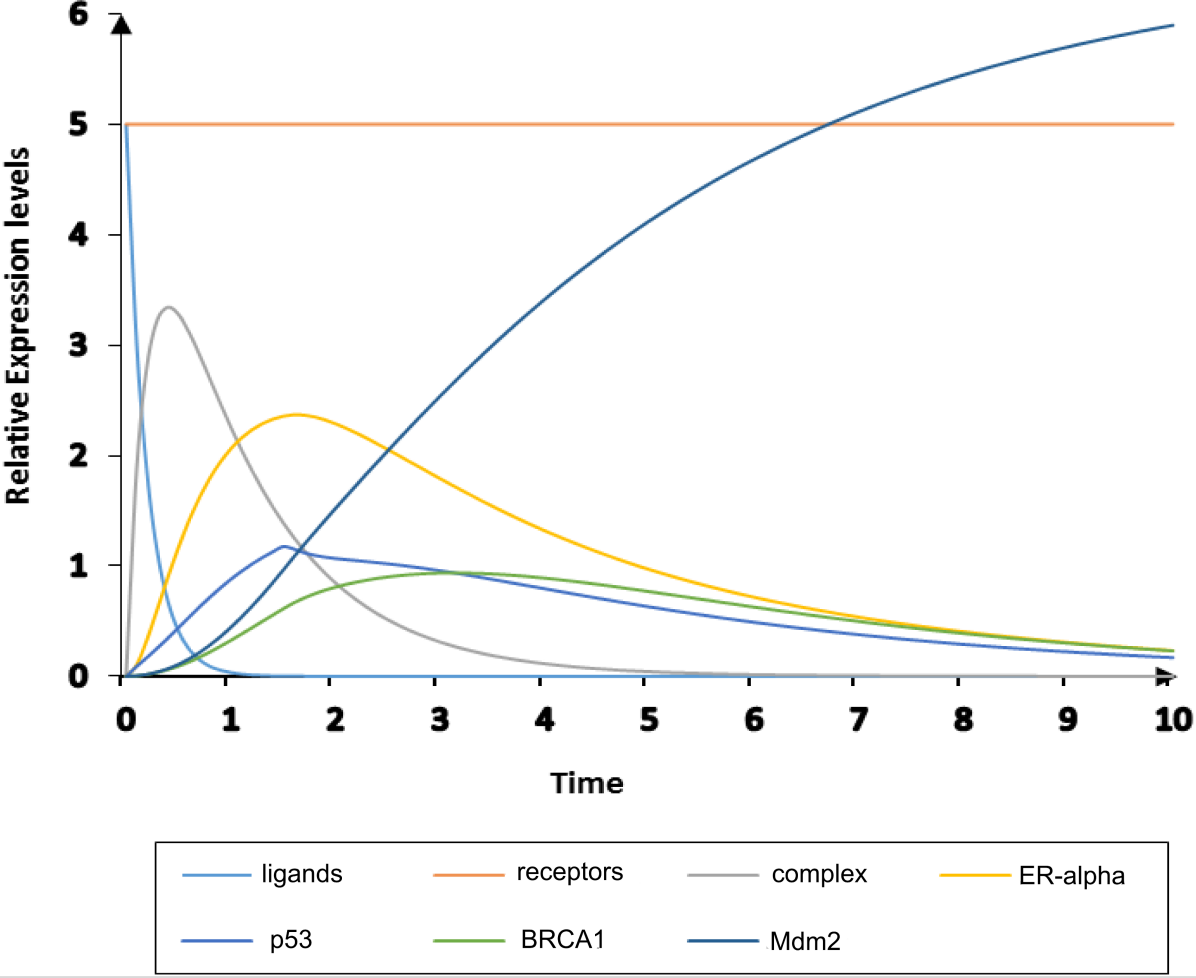
Simulation of homeostatic HPN model. The simulated graph shows time on *X*-axis and relative expression levels of entities on *Y*-axis. The homeostatic behavior of ER-*α* associated BRN is observed by the over-expression of TSG such as Mdm2 (navy) which down-regulates the activity of ER-*α* (yellow).

**Figure 9:**
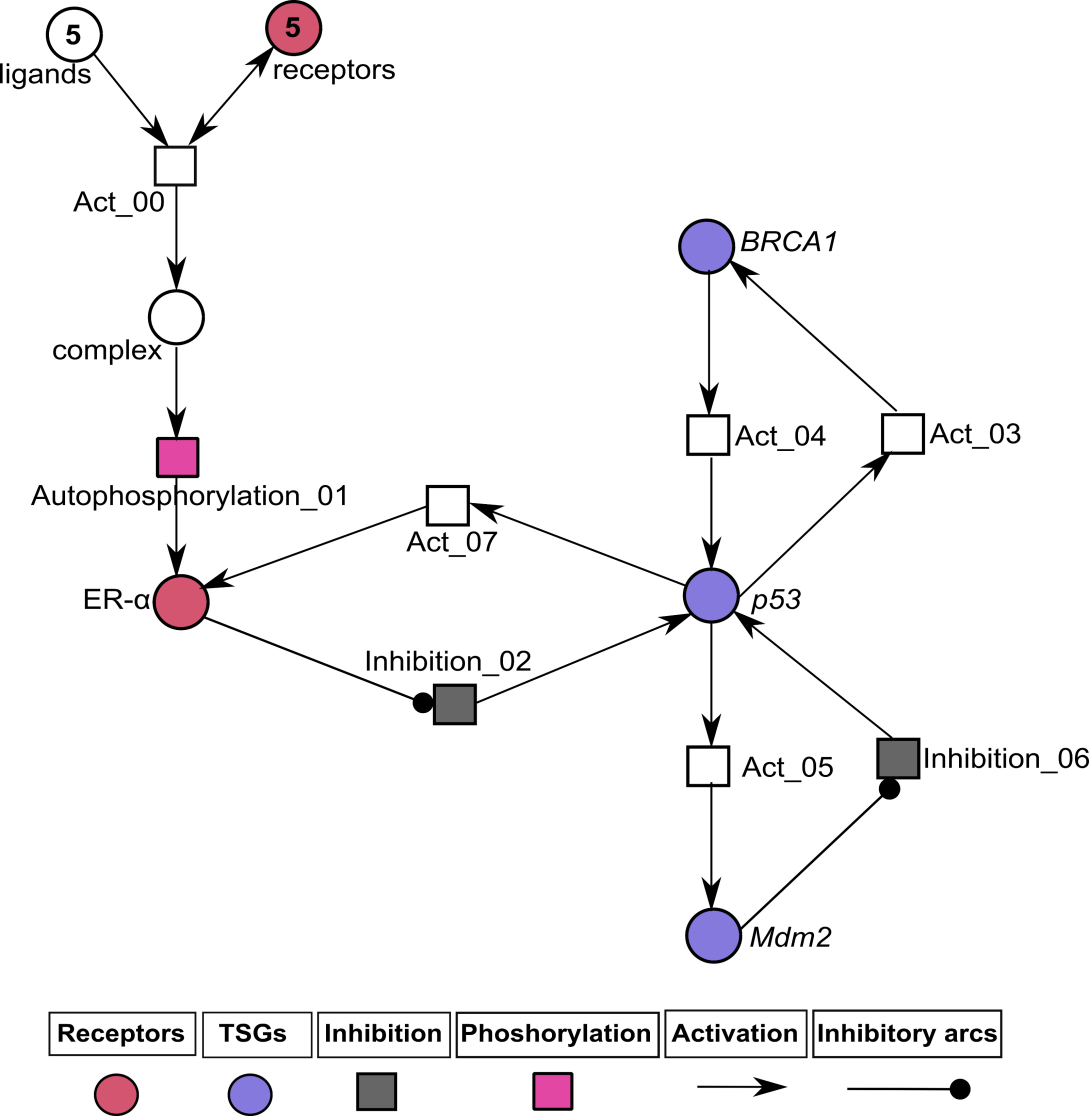
Illustration of the pathological pathway of ER-*α* associated HPN. In this PN circle represent standard places which explained the behavior of ligands (IGF-1, EGF), membrane and hormonal receptors (IGF-1R, EGFR and ER-*α*) and TSGs (BRCA1, p53 and Mdm2) and the squares represent continuous transitions to demonstrate the processes of activation, inhibition and phosphorylation. Directed arrows represent activation signal coming from standard places and going towards continuous transitions. Inhibitory arcs represent inhibition signal which stops signal coming from standard places towards continuous transitions. The rate of mass action for all continuous transitions is taken as 1. The ligands (IGF-1, EGF) and the membrane receptors (IGF-1R/EGFR) are given with an arbitrary token number of 5.

**Figure 10:**
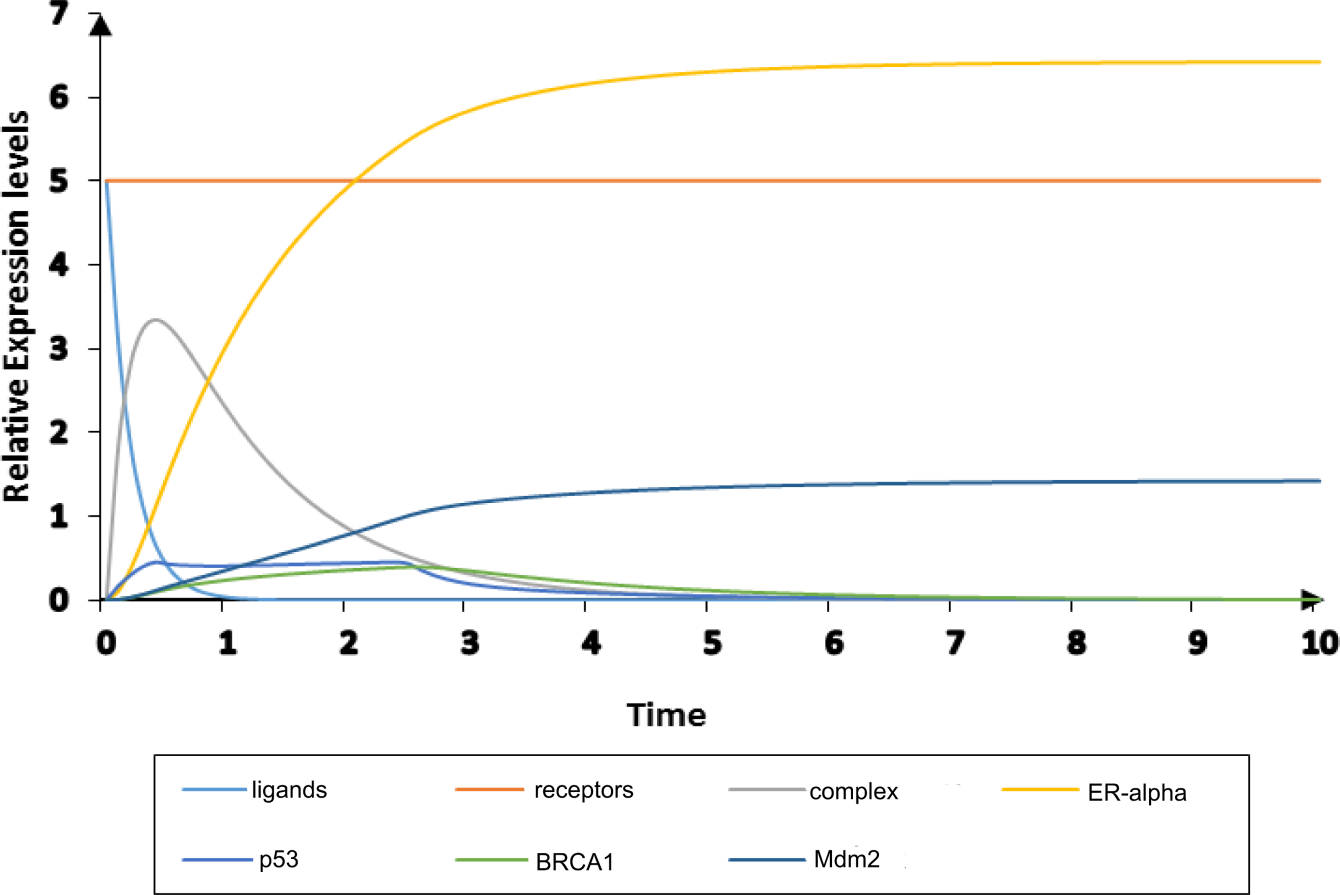
Simulation of diseased HPN model. The simulated graph shows time on *X*-axis and relative expression levels of entities on *Y*-axis. The pathological behavior of ER-*α* associated BRN is observed by the down-regulate expressions of TSGs; p53, BRCA1 and Mdm2 (cyan, green and navy) with relatively increased the activity of ER-*α* (yellow).

The PN model of BC metastasis was constructed to observe the time-dependent behaviors of key proteins of the BRN (given in ‘Construction of the ER-*α* associated BRN’). The HPN analysis was performed to reveal continuous dynamics of homeostatic and pathological conditions of the ER-*α* associated network. Two PN models and their simulations of ER-*α* were constructed (1) one to represent the normal behavior (given in Figs. 7 and 8) and other (2) to represent pathogenesis (Figs. 9 and 10) to evaluate the role of ER-*α* in BC. Both HPN models consist of 7 places, 8 transitions and 18 edges. The homeostatic ER-*α* associated HPN model (Fig. 7) has a positive feedback loop between *p*53 and ER-*α* which is switched on through the binding of ligands (IGF-1/EGF) with receptors (IGF-1R/EGFR) (Angeloni et al., 2004). This binding of receptors with ligands leads towards phosphorylation of kinases PI3K and AKT that ultimately cause up-regulation of ER-*α* (Kang et al., 2012a). The up-regulate expression of ER-*α* is controlled by the negative feedback interaction of TSG such as Mdm2.

The simulation results demonstrate in Fig. 8 of ER-*α* associated HPN model under homeostatic conditions. It shows the dynamical behavior of each entity that can be seen clearly through simulation graph plotted relative to the expression level of entities with respect to time. It has been observed that feedback regulation of *Mdm2* limits over-expression of ER-*α* by the inhibitory effect of TSGs (Berger et al., 2012; Ma et al., 2010) represented by yellow sigmoidal curve for ER-*α* (low level of expression) and cyan, green and navy sigmoidal curves for TSGs (high level of expression) to maintain the stability of the cellular environment. The continuous signaling of TSGs maintains the constant level of receptors (IGF-1R/EGFR) represented by an orange colored line. It shows how TSGs (p53, BRCA1 and Mdm2) perform the function of BC suppression (Bailey et al., 2012; Berger et al., 2012; Kim, Burghardt & Barhoumi, 2011; Ma et al., 2010; Sotiriou et al., 2003). The significantly increased expression of *Mdm2* is observed by the transcriptional activation of p53 gene (Liu et al., 2009; Ma et al., 2005; Miller et al., 2005). *p*53 down-regulates the expression of hormonal receptor (ER-*α*) through the stimulation of *BRCA1* and *Mdm2.* The basal level of *p*53 is retained through a negative feedback control of *Mdm2* upon *p*53 under homeostatic condition.

The pathological ER-*α* associated HPN was constructed to demonstrate the inhibitory effect of ER-*α* on *p*53 shown in Fig. 9. As TSGs are down-regulated during pathogenesis by the hyperactivity of ER-*α* so the processes of cell cycle regulation, DNA damage and repair are considerably suppressed (Bailey et al., 2012; Kang et al., 2012a; Liu et al., 2009; Miller et al., 2005; Surmacz & Bartucci, 2004; Sotiriou et al., 2003). The up-regulate expression of ER-*α* is achieved by the transactivation and phosphorylation of ligands (IGF-1/EGF) which binds to receptors (IGF-1R/EGFR) given by a token number of 5. ER-*α* is closely associated with cancer biology, especially with the development of tumor in BC (Alluri, Speers & Chinnaiyan, 2014). So it is important to study the mechanism of ER-*α* associated signaling pathway is controlled by the inhibition of complex (ligands binding with receptors) to obtain new insight into the treatment of BC.

The pathological conditions of ER-*α* associated HPN were simulated to observe the expression levels of entities with respect to time, given in Fig. 10. The mutated behavior of TSGs can be clearly seen in the simulation graph where sigmoidal curves for *BRCA1, p53* and *Mdm2* are represented by cyan, green and navy colors, respectively. Likewise, ER-*α* is produced at constant pace (represented at the expression level of 5) with mutated behaviors of TSGs which stimulates the activity of IGF-1R and EGFR receptors (represented by orange colored line). The high level of IGF-1R in ER-positive (ER+) BC cells is attributed to the carcinogenic cellular proliferation (Yerushalmi et al., 2012). The gene expression profile of basal cancer subtypes ER-PR-HER2 has low expression of ER-related genes and high expression of basal marker than luminal cancer (Perou et al., 2000; Sorlie et al., 2001; Sotiriou et al., 2003). The phosphorylation of receptors carried out by ligands IGF-1/EGF is involved in the development of BC pathogenesis (Kang et al., 2012b; Levin, 2001) depicted by blue colored curve. Various epidemiological studies have revealed that the increased level of IGF-1 is associated with higher risk of other malignancies such as prostate, colorectal and postmenopausal BC (Giovannucci, 2001; Kang et al., 2012b; Soulitzis et al., 2006). Previous evidences shows the over-expression of IGF-1R and EGFR in various types of breast tumours such as luminal and basal cancer subtypes (Perou et al., 2000; Sorlie et al., 2001; Sotiriou et al., 2003; Yerushalmi et al., 2012). Trastuzumab is a monoclonal antibody used in targeted therapy to prevent another subtype of BC which is HER2-positive (HER2+) (Lu et al., 2001). The activity of trastuzumab is disrupted by the over-expression of both IGF-1R and EGFR in BC cells that overexpress HER2 (Gallardo et al., 2012). Our results also suggest that inhibition of the carcinogenic effect of IGF-1R and EGFR in ER-*α* signaling pathway tend to reduce BC cell proliferation and metastasis.

#### Comparison of homeostatic and disease HPN models

**Figure 11:**
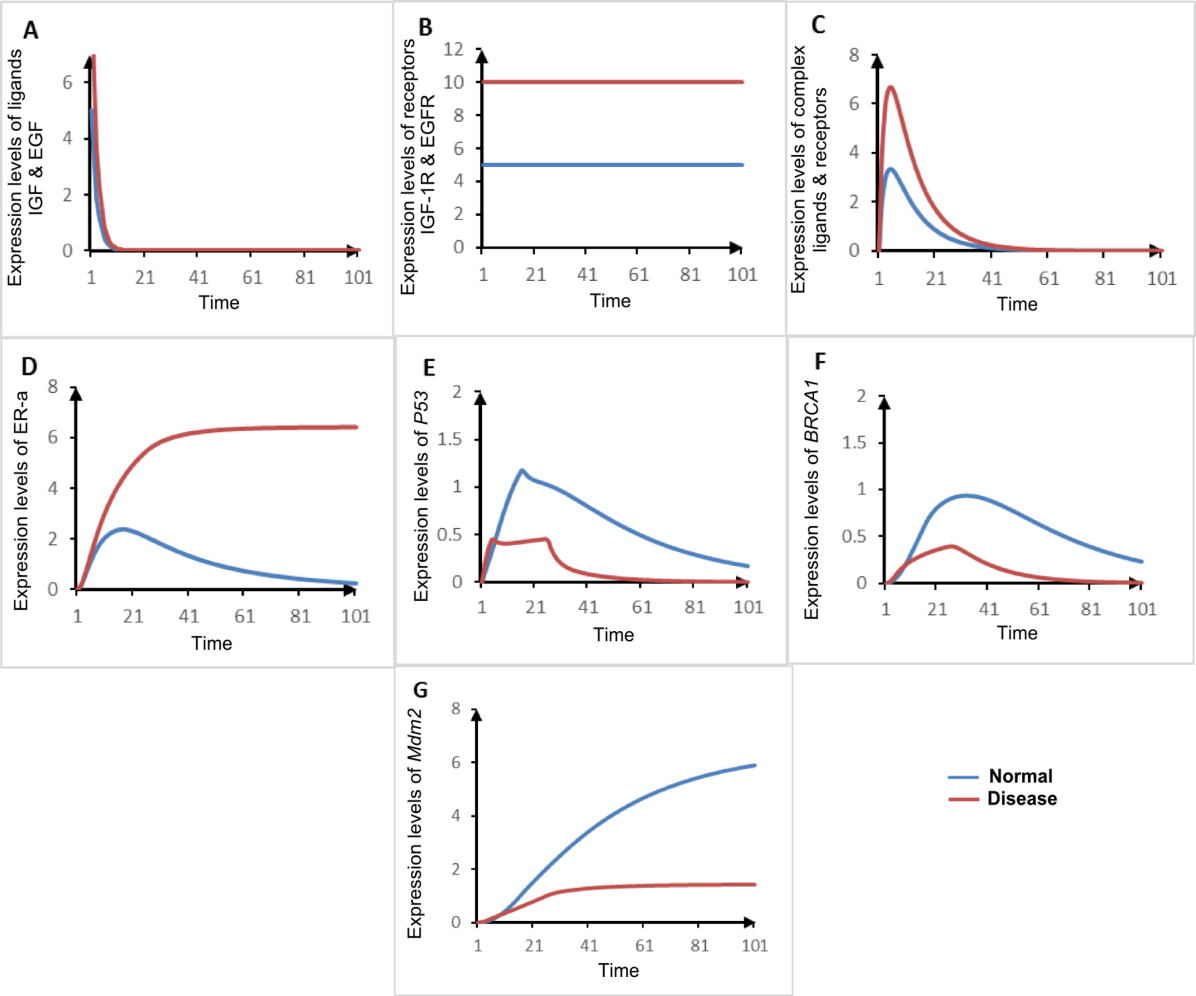
Comparison of simulated graphs of both homeostasis and disease ER-*α* associated HPN models. The *X*-axis shows the time unit while *Y*-axis shows the expression level of each entity under homeostasis and disease conditions of HPN models (Figs. 10 and 8). The blue line/curve represents the homeostatic behaviors and the brown line/curve represents the expression levels of mutated behaviors of key proteins involved in ER-*α* associated pathway. Figs.11A–11G represents the relative change in activity levels of ligands (IGF-1/EGF), receptors (IGF-1R/EGFR), complex, ER-*α* and TSGs (BRCA1, p53, and Mdm2) before and after mutations to be occurred.

The comparison of the dynamical behavior of proteins involved in ER-*α* associated signaling pathway in homeostasis and pathological conditions in BC has been performed in accordance with the biological observations as shown in Table 2 and Fig. 11, respectively. The differences in simulation graphs represent the relative expression level of each entity under the state of homeostasis (represented by blue color) and pathogenesis (represented by brown color). The change in interaction is based on our interpretation of the results from the BRN modeling. Our results reproduced recent wet-lab findings previously performed to deregulate BC pathogenesis by using genome/protein wide expression and sequence analysis. In Figs.11A–11F were brown colored line/curve represents suppressed activity level of TSGs by the up-regulation of ER-*α* (Zhang et al., 2014) and blue colored line/curve represents the controlled levels of ER-*α* through the stimulation of TSGs (Berger et al., 2012).

**Table 2:** Comparison of expression levels of entities of both homeostasis and disease ER-*α* associated HPN simulation with respect to literature search. The positive sign (+) indicates the up-regulate expression, double positive (++) sign indicates the over-expression and a negative sign (−) indicates the down-regulate the expression levels of entities.

Genes	Homeostasis		Disease	
	Literature	Simulation	Literature	Simulation
Ligands IGF-1/EGF	− Surmacz & Bartucci (2004)	−	+ Kang et al. (2012a)	+
Receptors IGF-1R/ EGFR	− Surmacz & Bartucci (2004)	−	++ Ouban et al. (2003); Surmacz & Bartucci (2004); Taunk et al., (2010)	+
ER-*α*	− Zhang et al. (2014)	−	++ Bailey et al. (2012); Surmacz & Bartucci (2004); Liu et al. (2006)	++
BRCA1	+ Ma et al. (2010)	+	− Kang et al. (2012a); Rosen et al. (2003)	−
p53	+ Berger et al. (2012); Miller et al. (2005)	+	− Angeloni et al. (2004); Bailey et al. 2012; Liu et al. (2009)	−
Mdm2	+ Berger et al. (2012)	++	− Kim, Burghardt & Barhoumi (2011)	−

Comparison of homeostatic and pathogenic behaviors (through a simulation graph of each entity given in Fig. 11) exhibits strong co-relation of our results with literature given in Table 2. This shows the similar expression levels of entities obtained through qualitative modeling and literature except levels of *Mdm2*, IGF-1R and EGFR. The levels of ligands, receptors and ER-*α* are down-regulated in homeostasis, represented by a negative sign (−) as compared to pathogenesis (Surmacz & Bartucci, 2004; Zhang et al., 2014). Under pathological conditions, the rate of production of ER-*α* is over-expressed given by a double positive sign (++) as observed in both simulation and previous studies (Surmacz & Bartucci, 2004; Zhang et al., 2014). The up-regulated expression level of TSGs (BRCA1, p53 and Mdm2) observed under homeostatic conditions is represented by a positive sign (+) (Berger et al., 2012; Ma et al., 2010). On the basis of simulation results, the over-expression of *Mdm2* is suppressed by the phosphorylation of AKT kinases. We assume that variables in DNA damage whose synthesis depends on ionizing radiation (IR) and oxidative stress (OS) which independently shortens the half-life of *Mdm2* (Gueven et al., 2001; Yi, Kang & Bae, 2014). The autophosphorylation of AKT and ERK can, in turn, activate downstream mediator ER-*α*, resulting in up-regulation of IGF-1R and EGFR expressions. The BRN constructed in this paper is based on multiple independent datasets obtained from previous studies which showed expression of interlinked gene/protein through genome wide arrays, DNA copy number, sequencing, immunohistochemistry, micro RNA and reverse phase protein analysis (Bailey et al., 2012; Caldon, 2014; Kang et al., 2012a; Kang et al., 2014; Liao et al., 2014; Malaguarnera & Belfiore, 2014; Moerkens et al., 2014; Cancer Genome Atlas Network, 2012; Pollak, 1998; Sotiriou et al., 2003). In healthy individuals, TSGs complement each other to maintain homeostasis in the body. Any mutation in TSGs carries with it a high risk of developing cancer in estrogen responsive tissues (breast and ovarian) along-with over-expression of ER-*α* (Angeloni et al., 2004; Kim, Burghardt & Barhoumi, 2011; Liu et al., 2009; Rosen et al., 2003; Savage & Harkin, 2015). The treatment of ER+ metastatic BC using an antagonist in combination with drugs could lead to the regulation of *p*53 mediated apoptotic response (Bailey et al., 2012).

In ER+ BC treatment, strategies aimed at eliminating estrogen sources were developed few decades ago. *Tamoxifen* was the first such targeted therapy, also known as selective estrogen receptor modulator (SERM) that inhibits estrogen in many tissues. Further, *tamoxifen* is used for treatment of all stages of BC including adjuvant therapy, metastatic disease, and even as a preventive measure (Macgregor & Jordan, 1998). SERM binds to the ER and prevents estrogen from binding the ligand; however, dimerization and DNA binding followed by inhibition of transcription occur. SERM holds the ER in an inactive conformation and prevents the recruitment of co-activators (Paige et al., 1999). The common limitation is the development of resistance against *tamoxifen* in the advanced stages of BC. One mechanism of resistance to *tamoxifen* is increased through growth factor signaling pathways, such as the IGF pathway (Gallardo et al., 2012; Knowlden et al., 2005; Zhao & Ramaswamy, 2014). In addition to SERMs, aromatase inhibitors, such as *exemestane, anastrozole*, and *letrozole* deprive target tissues of ligand for ER which results in the inhibition of this pathway (Pietras, 2006; Van Asten et al., 2014). Steroidal anti-estrogens such as fulvestrant prevent ER dimerization, DNA binding and hence loss of receptor from cells (Agrawal et al., 2016; Osborne, Wakeling & Nicholson, 2004; Wakeling, Dukes & Bowler, 1991).

Studies show that estrogen can regulate IGF signaling and activate its downstream pathways by increasing the expression of both IRS-1 and IGF-1R in BC cells (Fagan & Yee, 2008; Lee et al., 1999). Our result obtained by using the tools GENOTECH, SMBioNet and SNOOPY have suggested that IGF-1R, EGFR and ER-*α* signaling pathways are actively involved in the progression of BC metastasis and they should be targeted together for its treatment. Our findings suggested an improved strategy for a combined drug therapy which confirms the results of few previous studies in which inhibition of both IGF-1R and EGFR have induced apoptosis by blocking phosphorylation of AKT and NF*κ*B. Previous studies have shown the inhibition of IGF-1R and EGFR in signaling pathways at multiple levels in adrenocortical, prostate, head and neck cancers (Lee et al., 2016; Raju et al., 2015; Xu et al., 2016). Commercially available inhibitors (*NVP-AEW541, gifitinib* and *erlotinib*) used against IGF-1R and EGFR significantly enhance anti-tumour efficacy for treatment of adrenocortical carcinoma (Baselga et al., 2005; Dickler et al., 2009; Hartog et al., 2012; Von Minckwitz et al., 2005; Xu et al., 2016). Therefore the combination of these commercially available inhibitors with systemic drugs (*tamoxifen*, *trastuzumab and fulvestrant*) should be used in the treatment of different clinical BC subtypes. In conclusion, blocking both EGFR and IGF-1R can inhibit estrogen stimulation of BC cells and blockade of ER-*α* signaling pathway can inhibit IGF-mediated mutagenesis.

## Conclusion

*In-silico* approaches (such as computational drug designing or computational gene-gene interaction modeling) are used to find the inhibitory targets which save our time and energy by reducing laborious trial and error methods. The kinetic logic, graph theoretical and model checking formalisms offer biologists the exciting prospect of being able to test hypotheses regarding network dynamics. It is imperative for scientists to understand changes in the expression levels of genes and proteins at cellular level. This is typically achieved through costly experimental techniques. However, it is possible to derive logical networks that can mimic the behavior of key drivers of transformation in the cell without extensive wet-lab experimentation. We have successfully deployed techniques encompassing the important features of ER-*α* associated BRN in response to various alterations in the stimuli or genetic changes in cancer cells. Based on previous findings and our model, we suggest that inhibiting ER-*α*, IGF-1R and EGFR together can be used for BC treatment. Therefore, *in-silico* approaches are used here to potentiate therapeutic target in combined strategies to improve clinical outcome in the future.

## Supplemental Information

10.7717/peerj.2542/supp-1 Supplemental Information 1 Supplementary file of Formal Modeling of ER-alpha

## Additional Information and Declarations

### Data Availability

The following information was supplied regarding data availability: The raw data has been supplied as  Supplemental Information 1.
