# Supplementary material for: Formal modeling and analysis of ER-α associated Biological Regulatory Network in breast cancer
Source: PeerJ. 2016 Oct 20;4:e2542. doi: 10.7717/peerj.2542 (PMC5075711; doi:10.7717/peerj.2542)
Supplement: Supplemental Information 1 [file peerj-04-2542-s001.pdf]

Supplementary file

Input file of (Estrogen Receptor-Alpha) ER-α associated network:

A suitable selected model has been generated in this file of SMBioNet software. It has four steps such as Variables (VAR), Regulation (REG), Parameterization (PARA) and Computational Tree Logic (CTL).

1st step: Set of Variables (V) is defined as VAR of Biological Regulatory Network (BRN) as follows:

VAR

IGF-1R/EGFR = 0 1;
BRCA1 = 0 1;
ER-α = 0 1;
p53 = 0 1;
Mdm2 = 0 1

These five set of variables evolve into the interval {0, 1}. All possible number of arcs are 2^5=32 in state graph.

2nd step: The set of multiplexes (M) and edges (E_D) defines the regulatory graph (REG) of ER-α associated network is described as follows:

REG

BRCA1[(*BRCA1*<1)]=> IGF-1R/EGFR, ER-α;
BRCA1[(*BRCA1*>=1)]=> *p53*;
p53[(*p53*>=1)]=> ER-α;
p53[(*p53*>=1)]=> *BRCA1*, *Mdm2*;
Mdm2[(*Mdm2*<1)]=> *p53*;
IGF-1R/EGFR[(IGF-1R/EGFR>=1)]=> ER-α;
ER-α[(ER-α<1)]=> *p53*

In this parameterization of REG, where "<" indicates the inhibition and ">" indicates the activation of BRN. In case of *BRCA1* inhibits the activation of IGF-1R/EGFR and ER-α. In the absence of inhibitor *p53*, activation of the receptors IGF-1R and ER-α. *p53* activates *BRCA1* and *Mdm2*. The BRN of ER-α associated pathway is represented in introduction Figure (1) where the targeted genes are IGF-1R/EGFR and ER-α while Tumor suppressor genes TSGs (BRCA1, p53 and Mdm2) are multiplexes.

3rd Step: Set of some possible parameters is defined as Parametric (PARA) constraints, which selects the reduced parameters. We constraint the parameter in BRN such as K_$_{p53+BRCA1+\text{ER-}\alpha}$ = ({Φ}) in the integer interval [0, 1].

Para

K_*p53*+*BRCA1*+ER-α = 1;

Parametric constraints are important for the selection of suitable possible parameter.

4th step: CTL is a temporal operator which defines the dynamical properties of the system. The syntax and semantic of CTL is discussed at earlier subsection. CTL formula is define in SMBioNet as follows:

CTL

((IGF-1R/EGFR=0&ER-α=0&*BRCA1*=0&*p53*=0&*Mdm2*=0)>EX (EF(IGF-1R/EGFR=0&ER-α=0&*BRCA1*=0&*p53*=0&Mdm2=0))) &((ER-α=0)>EF(ER-α=1))&((IGF-1R/EGFR=0&ER-α=0& *BRCA1*=0&*p53*=0&*Mdm2*=0)>EF(AG(IGF-1R/EGFR=1&ER-α=1)))

It has three sections in conjunction of CTL formula. In the first section of this equation consider that a successor from which there exist a path where in future the set of all the initial states hold. If there is any mutation like DNA damage and radiation occurs then increase the level of ER-α in the absence of p53, BRCA1 and Mdm2 gene. While in the next section of CTL it is determining that there exist a path in future where persistently ER-α is zero. The third section determines that there should exist one path in future where all paths and states globally IGF-1R/EGFR and ER-α at the level of one.

Output file

Output file has consistent logical parameters of selected model verify by CTL formulism in BRN. There were five models generated in input file while one of the selected model is discussed in output file. It has reduced mathematical 32 parameters with the infinite known properties of system is determines as follows:

K_IGF-1R/EGFR = 1
K_*BRCA1* = 0
K_*BRCA1+p53* = 1
K_ER-α = 0
K_ER-α+IGF-1R/EGFR = 1
K_ER-α+*p53* = 1
K_ER-α+IGF-1R/EGFR+*p53* = 1
K_*p53* = 0
K_*p53+BRCA1* = 1
K_*p53*+ER-α = 0
K_*p53+Mdm2* = 0
K_*p53+BRCA1*+ER-α = 1
K_*p53*+ER-α+*Mdm2* = 1
K_*p53+BRCA1+Mdm2* = 1
K_*p53+BRCA1*+ER-α+*Mdm2* = 1
K_*Mdm2* = 0
K_*Mdm2+p53* = 1

These parameterizations are consistent with possible behavior of biological entities rather than inconsistent manner.
